# A Two-Stage Method Based on Multiobjective Differential Evolution for Gene Selection

**DOI:** 10.1155/2021/5227377

**Published:** 2021-12-20

**Authors:** Shuangbao Song, Xingqian Chen, Zheng Tang, Yuki Todo

**Affiliations:** ^1^Aliyun School of Big Data, Changzhou University, Changzhou 213164, China; ^2^Faculty of Engineering, University of Toyama, Toyama-shi 930-8555, Japan; ^3^Faculty of Electrical and Computer Engineering, Kanazawa University, Kanazawa-shi 920-1192, Japan

## Abstract

Microarray gene expression data provide a prospective way to diagnose disease and classify cancer. However, in bioinformatics, the gene selection problem, i.e., how to select the most informative genes from thousands of genes, remains challenging. This problem is a specific feature selection problem with high-dimensional features and small sample sizes. In this paper, a two-stage method combining a filter feature selection method and a wrapper feature selection method is proposed to solve the gene selection problem. In contrast to common methods, the proposed method models the gene selection problem as a multiobjective optimization problem. Both stages employ the same multiobjective differential evolution (MODE) as the search strategy but incorporate different objective functions. The three objective functions of the filter method are mainly based on mutual information. The two objective functions of the wrapper method are the number of selected features and the classification error of a naive Bayes (NB) classifier. Finally, the performance of the proposed method is tested and analyzed on six benchmark gene expression datasets. The experimental results verified that this paper provides a novel and effective way to solve the gene selection problem by applying a multiobjective optimization algorithm.

## 1. Introduction

Gene selection is an important issue in bioinformatics [1]. A gene is the basic functional unit of heredity. Gene expression is the process in which the instructions encoded in genes are used to synthesize gene products [2] such as proteins. Then, the gene products dictate cellular function. Therefore, abnormal gene expression is usually correlated with different types of disease, such as cancer [3]. Usually, many diseases correspond to unique gene expression profiles that can be revealed by DNA microarray technology [4]. Typically, microarray data corresponding to a certain disease consist of a set of biological samples. From each sample, the expression of thousands of genes at each position can be measured. As a result, microarray data are usually in the form of a matrix. However, it is not an easy task for researchers to check which genes are responsible for a given disease because of the high dimensionality of microarray data. Thus, determining how to select the most significant genes effectively for further analysis becomes urgent and vital.

The gene selection problem is intrinsically a feature selection problem with high-dimensional features and small sample sizes. Since the gene expression data can be labeled (whether the sample is malignant or not), partially labeled, or unlabeled, three categories of methods are applied to solve the gene selection problem in the literature [5]: supervised, semisupervised, and unsupervised feature selection methods. Because labeled data are the most common types of data in reality, supervised feature selection methods are the most widely used and most practical methods for the gene selection problem. We refer to feature (gene) selection methods as supervised feature (gene) selection methods in the following context.

In the field of machine learning, feature selection, also known attribute selection, is defined as the process by which the best subset of relevant features is selected from a large set of features [6], and the performance of classifiers is assuredly improved by the optimal feature subset when compared with the utilization of all features. However, it is difficult to execute feature selection by retaining relevant features and removing irrelevant and redundant features. There are two main obstacles in feature selection. First, the size of the search space is quite large. Given a dataset with *n* features, there are 2^*n*^ subsets (solutions) [7]. Specifically, as big data continues to grow [8], *n* becomes increasingly large. Thus, in most cases, an exhaustive search for feature selection is impossible. Second, the feature interaction problem makes feature selection complex. For example, a feature as a single entity is irrelevant to the target, but when combined with another feature, it may become significantly relevant. In fact, there are many interaction patterns among features. As a result, “the *m* best features are not the best *m* features” [9]. Therefore, the performance of a feature selection method depends on two key factors: (1) effective evaluation criteria to measure the quality of a feature subset and (2) an efficient search strategy to explore the large search space [10].

Regarding evaluation criteria, feature selection methods can be roughly classified into two categories: filter methods and wrapper methods [10]. The main difference between them is that wrapper methods use a classifier to evaluate a feature subset, while filter methods do not. Filter methods are independent of any classifier and focus on the intrinsic characteristics of the dataset. The common metrics used in filter methods are correlation [11] and mutual information [12]. Specifically, the filter methods examining each feature separately are considered univariate. They ignore the feature interaction problem and lead to the redundancy of feature subsets. Thus, multivariate filter methods such as minimum redundancy-maximum relevance (mRMR) [13] are considered better choices. Wrapper methods select discriminative feature subsets to improve the classification performance. Most popular classifiers can be incorporated into wrapper methods, e.g., the naive Bayes (NB), K-nearest neighbors, support vector machine, and neural network [14]. It has been generally regarded that filter methods are usually considered faster, but their accuracy is relatively lower. Wrapper methods are the opposite of filter methods because they need to consider the computational costs of the involved classifiers. Thus, combining them as a hybrid method is an alternative and promising method for feature selection problems, especially for the gene selection problem [15].

There are two main categories of search strategies applied in feature selection. The first category is sequential search. Sequential forward selection and sequential backward selection [16] are considered conventional methods but suffer from the “nesting effect” [17] because only one feature is added or removed at a time. The second category is a randomized search strategy that starts by randomly selecting some features and then executing a heuristic search. It has been verified that these methods based on randomized search are better than the methods based on sequential search because they can escape local optima more easily [10]. Specifically, applying evolutionary computation (EC) techniques such as genetic algorithms (GAs) [18], particle swarm optimization (PSO) [19, 20], and differential evolution (DE) [21, 22] to feature selection has raised the attention of researchers in recent years.

Regarding the gene selection problem, numerous methods based on EC techniques have been proposed in the literature [5]. These pertinent experiments have shown that EC techniques can achieve very competitive performance compared with traditional methods. For example, Mohamad et al. proposed an improved binary PSO as a wrapper method and obtained positive results [23]. Shreem et al. proposed a Markov blanket-embedded harmony search algorithm as a wrapper method to solve the gene selection problem [24], and Elyasigomari et al. proposed a filter method based on the cuckoo optimization algorithm and shuffling [25], where a clustering technique was involved. In addition, a modified artificial bee colony algorithm was applied to solve the gene selection problem in the work of Alshamlan et al. [26], where the search method was enhanced by combining two EC algorithms. Note that most current methods based on EC techniques treat the gene selection problem as a single-objective optimization problem. On the other hand, recent work [22, 27] suggests that multiobjective optimization techniques are alternatives for solving the gene selection problem. This is because the single objective to multiobjective transformation can lead to improvements in the search strategy and evaluation criteria; thus, more competitive results can be obtained. However, to the best of the authors' knowledge, employing an effective multiobjective differential evolution (MODE) approach to address the gene selection problem has not yet been well explored.

Thus, in this study, a two-stage method based on multiobjective optimization is proposed. The first stage included a multivariate filter method where three objective functions referring to mutual information are incorporated. The second stage included a conventional wrapper method involving the NB classifier. The number of selected features and the classification error are incorporated as the two objective functions in this stage. In addition, both stages employ the same search strategy: a well-designed MODE. Finally, six benchmark datasets are used to test and analyze the performance of the proposed method. The experimental results are statistically compared with those of five widely used feature selection methods.

The remainder of the paper is organized as follows.  Section 2 introduces three important concepts: multiobjective optimization, differential evolution, and mutual information.  Section 3 describes the proposed method.  Section 4 provides the experimental results and analysis. Finally, Section 5 draws the conclusion of this paper.

## 2. Materials

### 2.1. Multiobjective Optimization Problem

Many real-world problems involve multiple conflicting objectives that should be optimized simultaneously [28]. A MOOP is a multiobjective minimization problem that involves more than one objective function to be optimized, and it can be mathematically stated as follows:

(1) $$\begin{array}{c} \mathrm{minimize}\;f\left(x\right)=\left(f_{1}\left(x\right),f_{2}\left(x\right),\ldots ,f_{k}\left(x\right)\right)\\ \text{s}.\text{t}.\;x=\left(x_{1},x_{2},\ldots ,x_{n}\right)\in \Omega , \end{array}$$

where **x** is the *n*-dimensional decision vector and Ω is the decision space. **f** : Ω⟶*R*^*k*^ consists of *k*(*k* ≥ 2) real-valued objective functions *f*_1_(**x**), *f*_2_(**x**),…, and *f*_*k*_(**x**). In normal cases, there is no solution that can optimize all the objective functions because of the conflicts among these objectives. Four important definitions referring to MOOPs are given as follows.

**Definition 1 .**

(Pareto dominance). Let **a**=(*a*_1_, *a*_2_,…, *a*_*n*_) and **b**=(*b*_1_, *b*_2_,…, *b*_*n*_) be two vectors. **a** is said to dominate **b**, represented as **a**≺**b**, if

(2) $$\begin{array}{c} \left(1\right)\;\forall i\in \left\{1,2,\ldots ,k\right\},\;f_{i}\left(a\right)\leq f_{i}\left(b\right),\\ \left(2\right)\;f\left(a\right)\neq f\left(b\right). \end{array}$$

**Definition 2 .**

(Pareto optimal solution). For a given MOOP, a vector **x**^*∗*^ ∈ Ω is called the Pareto optimal solution if

(3) $$\begin{array}{c} \neg \exists x^{\prime }\in \Omega ,\;x^{\prime }≺\;x^{*}. \end{array}$$

**Definition 3 .**

(Pareto optimal set). All Pareto optimal solutions compose the Pareto optimal set *P*, which can be described as follows:

(4) $$\begin{array}{c} P=\left\{x^{*}\in \Omega |\neg \exists \;x^{\prime }\in \Omega ,x^{\prime }≺x^{*}\right\}. \end{array}$$

**Definition 4 .**

(Pareto front). The image of the Pareto optimal set is called the Pareto front *PF*, which is composed of objective vectors and is defined as follows:

(5) $$\begin{array}{c} PF=\left\{f\left(x\right)\;|\;x\in P\right\}. \end{array}$$

For a real-world MOOP, the Pareto optimal *P* is usually unreachable and infinite. Therefore, the goal of an optimization method [29–31] is to obtain an approximation of *P*, which is convergent and diverse in the objective space as much as possible. In addition, an excellent approximation of *P* is crucial for a decision maker to select the final solutions.

### 2.2. Standard Differential Evolution

**Figure 1:**
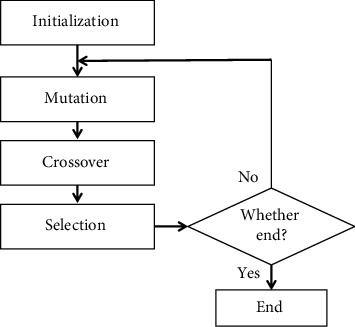
The flowchart of standard differential evolution.

DE is a simple but powerful stochastic optimization algorithm that was first proposed by Storn and Price in the 1990s [32]. Recent research has increased the efficiency for solving many real-world problems [33–35]. The characteristic of DE is using the difference between two candidate solutions to generate a new candidate solution. This algorithm is population based and works through a cycle of computational steps, which are similar to the steps employed in common evolutionary algorithms. The flowchart of standard DE is shown in Figure 1, and it can be separated into the following four stages: initialization, mutation, crossover, and selection.

DE optimizes a problem by maintaining a population of candidate solutions and evolving them with specific formulas within the search space. An individual, also called a genome, is represented as a vector forming a candidate solution for a specific problem as follows:

(6) $$\begin{array}{c} X^{\left(i\right)\left(t\right)}=\left(x_{1}^{\left(i\right)\left(t\right)},x_{2}^{\left(i\right)\left(t\right)},\ldots ,x_{d}^{\left(i\right)\left(t\right)}\right), \end{array}$$

where *d* is the dimension of the search space and **X**^(*i*)(*t*)^ represents the *i*th individual in the *NP*-sized population at generation *t*.

Initially, all individuals **X**^(*i*)(*t*)^, also called target vectors, are randomly initialized by restricting them in a problem-specific range. Then, standard DE starts its main loop. Every individual evolves in the following steps. First, for each individual **X**^(*i*)(*t*)^, the differential mutation operator works and generates a donor vector **V**^(*i*)(*t*)^=(*v*_1_^(*i*)(*t*)^, *v*_2_^(*i*)(*t*)^,…, *v*_*d*_^(*i*)(*t*)^) as follows:

(7) $$\begin{array}{c} V^{\left(i\right)\left(t\right)}=X^{\left(r_{1}\right)\left(t\right)}+F\left(X^{\left(r_{2}\right)\left(t\right)}-X^{\left(r_{3}\right)\left(t\right)}\right), \end{array}$$

where *F* is the mutation scale factor that controls the scaled difference and *r*_1_, *r*_2_, and *r*_3_ are three different integers, which are randomly chosen from the range [1, *NP*]. Note that the three integers must be different from the current index *i*.

Next, the trial vector **U**^(*i*)(*t*)^=(*u*_1_^(*i*)(*t*)^, *u*_2_^(*i*)(*t*)^,…, *u*_*d*_^(*i*)(*t*)^) is generated by crossing over the target vector **X**^(*i*)(*t*)^ and the donor vector **V**^(*i*)(*t*)^. A typical crossover mutation operation employed in standard DE is implemented by exchanging components between **X**^(*i*)(*t*)^ and **V**^(*i*)(*t*)^ as follows:

(8) $$\begin{array}{c} u_{j}^{\left(i\right)\left(t\right)}=\left\{\begin{array}{cc} v_{j}^{\left(i\right)\left(t\right)}, & \text{if}\left(r\leq Cr\;\mathrm{or}\;j=j_{r}\right),\\ x_{j}^{\left(i\right)\left(t\right)}, & \text{otherwise,} \end{array}\right. \end{array}$$

where *u*_*j*_^(*i*)(*t*)^ is the *j*th element of **U**^(*i*)(*t*)^ and *r* is a uniformly distributed random real number in [0,1]. *Cr* is the crossover rate that controls the probability of how many elements of **U**^(*i*)(*t*)^ are inherited from **V**^(*i*)(*t*)^. *j*_*r*_, which ensures that **U**^(*i*)(*t*)^ obtains at least one element from **V**^(*i*)(*t*)^, is a random integer in [1, *d*].

Then, the selection process is executed to update all individuals as follows:

(9) $$\begin{array}{c} X^{\left(i\right)\left(t+1\right)}=\left\{\begin{array}{cc} U^{\left(i\right)\left(t\right)}, & \text{if}\left(f\left(U^{\left(i\right)\left(t\right)}\right)\leq f\left(X^{\left(i\right)\left(t\right)}\right)\right),\\ X^{\left(i\right)\left(t\right)}, & \text{otherwise,} \end{array}\right. \end{array}$$

where *f*(·) is the single-objective function of DE.

Finally, the DE terminates when the stopping criterion is met.

### 2.3. Mutual Information

In information theory [36], the mutual information of two variables quantifies the mutual dependence between them. This metric measures the correlation between two variables powerfully and is not sensitive to the noise in sampling [37]. Given two continuous variables *x* and *y*, their mutual information can be defined as follows:

(10) $$\begin{array}{c} I\left(x;y\right)=\iint p\left(x,y\right)\log\frac{p\left(x,y\right)}{p\left(x\right)p\left(y\right)}\text{d}x\text{ d}y, \end{array}$$

where *p*(*x*) and *p*(*y*) are the probability density functions of *x* and *y*, respectively, and *p*(*x*, *y*) is the joint probability density function. Therefore, if two variables are strictly independent, their mutual information is equal to 0. Similarly, for two discrete variables *x* and *y*, mutual information has the following form:

(11) $$\begin{array}{c} I\left(x;y\right)=\sum_{x\in X}\sum_{y\in Y}p\left(x,y\right)\log\frac{p\left(x,y\right)}{p\left(x\right)p\left(y\right)}. \end{array}$$

Given two variables *x* and *y*, the range of the mutual information *I*(*x*; *y*) between them is [0, min{*H*(*x*), *H*(*y*)}], where *H*(·) is the function to calculate the entropy of a variable.

Although mutual information has been considered an excellent indicator to quantify the independence between two variables, its calculation is not easy because estimating probability density functions is a complex task. If two variables are discrete, the calculation of mutual information is straightforward by counting the samples in difficult categories to make the joint and marginal probability tables. However, if at least one of the two variables is continuous, the calculation becomes difficult. In this work, we use entropy estimation based on the K-nearest neighbor distance [38] to calculate mutual information.

## 3. Methodology

**Figure 2:**
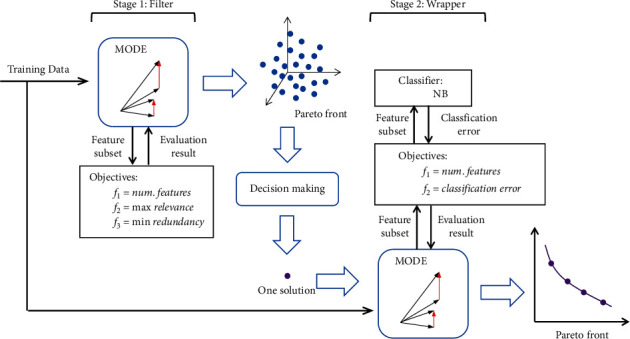
The flowchart of the proposed two-stage method based on MODE for gene selection.

In this section, the proposed two-stage method based on MODE is described.  Figure 2 illustrates the flowchart of the proposed method, which consists of two stages: a filter stage and a wrapper stage. In the latter stage, a novel wrapper method based on MODE is proposed. In addition, two single-objective wrapper methods based on DE are proposed in this stage. These two single-objective methods serve as the baseline to test the performance of the MODE-based wrapper method and help us investigate the following: (1) whether it is necessary to consider the number of selected features in the wrapper method and (2) whether the method based on multiobjective optimization outperforms the methods based on single-objective optimization.

### 3.1. Multiobjective Differential Evolution

Due to the effectiveness of DE for solving single-objective optimization problems, extending DE to solve MOOPs has attracted the interest of researchers in the literature [34]. Two important issues in extending DE into MODE need to be overcome. The first issue is how to order two candidate solutions. The solutions are straightforward to order when one solution dominates the other solution. However, if two candidate solutions do not dominate each other, an additional strategy to assign the complete order must be provided. Second, an effective scheme of maintaining a set of nondominated solutions during the optimization process is necessary. In contrast to single-objective optimization problems where only one global optimal solution is generated, the goal of MOOPs is to obtain a set of nondominated solutions. Therefore, the convergence and diversity of the set of nondominated solutions should be ensured. A widely used method is to adopt an external archive to couple with the current population [30].

The proposed MODE follows the framework of the standard DE, which is shown in Figure 1. The external archive stores the nondominated solutions that interact with the current population. In addition, the mutation operator and the selection operator, which are different from those of the standard DE algorithm, are modified. The key components of the proposed MODE are described below.

#### 3.1.1. External Archive

Adopting an external archive to store nondominated solutions is a common and effective method in numerous multiobjective evolutionary algorithms [39, 40]. Similarly, an archive *Arc* with limited size *N*_*a*_ is maintained in the optimization process of the proposed MODE. A solution *s* will be added into *Arc* if any one of the following criteria is met. (1) *Arc* is empty. (2) *Arc* is not full, and *s* is nondominated by any solution in *Arc*. (3) *s* dominates at least one solution in *Arc*. Note that in this case, these solutions dominated by *s* will be removed from *Arc*. (4) *Arc* is full, and *s* is nondominated with any one solution in *Arc*. In this extreme condition, *s* is first added into *Arc*, and a density estimation operation is executed to assign each solution a crowding distance value (see Section 3.1.2). Then, the solution in the most crowded region will be removed from *Arc*.

The archive *Arc* interacts with the current population in two aspects. First, the equation for generating a donor vector **V**^(*i*)(*t*)^ (see equation (7)) is modified by

(12) $$\begin{array}{c} V^{\left(i\right)\left(t\right)}=X^{\left(r_{arc}\right)\left(t\right)}+F\left(X^{\left(r_{2}\right)\left(t\right)}-X^{\left(r_{3}\right)\left(t\right)}\right), \end{array}$$

where **X**^(*r*_*arc*_)(*t*)^ is a solution that is randomly selected from the external archive *Arc* rather than the current population. This handling method is inspired by the standard mutation strategy in DE/best/1 [41]. **X**^(*r*_*arc*_)(*t*)^ can be regarded as one of the best solutions that are stored in archive *Arc*. Second, the selection operator (see equation (9)) of MODE is modified, which is illustrated in Algorithm 1, and the interaction between the current population and archive *Arc* will be enhanced. Since the updating scheme of archive *Arc* is based on crowded information, archive *Arc* will be updated in a timely manner at each iteration, and the convergence and diversity of these nondominated solutions in *Arc* can be ensured.

#### 3.1.2. Density Estimation

Many density estimation methods have been proposed in the literature [29, 30]. In our proposed method, a parameter-independent method called the crowding distance is used to assist Pareto dominance in assigning the complete order. The basic idea is that the degree of crowding of a solution in objective space is quantified by the distance between its neighbors. For a given solution *s* and an archive *Arc*, the crowding distance of *s* can be calculated by Algorithm 2. This method is similar to the method used in the nondominated sorting genetic algorithm-II (NSGA-II) [29], and the crowding distance of a solution is considered the perimeter of the cuboid formed by its neighbors.

#### 3.1.3. Parameter Control

The mutation scale factor *F* (see equation (7)) and the crossover rate *Cr* (see equation (8)) are the two main control parameters in DE. A well-tuned setting of *F* and *Cr* is crucial to the performance of DE [41]. However, determining how to set the suitable values of *F* and *Cr* is problem-specific. To select suitable parameters for *F* and *Cr*, we follow the idea of self-adaptive differential evolution (SaDE) [42] and use a self-adaptive strategy to control the two parameters in MODE.

The employed parameter control strategy is described as follows. At each iteration, a set of *F* values is regenerated from a normal distribution with a mean of *μ*=0.5 and a standard deviation of *σ*=0.3. Then, these *F* values are orderly applied in equation (12) to generate the donor vectors. In this way, both exploitation (small *F* values) and exploration (large *F* values) are ensured during the evolution process. Furthermore, the crossover rate *Cr* is gradually adjusted according to previous experience during the evolutionary process. Specifically, *Cr* is assumed to obey a normal distribution with a mean of *μ*=*Cr*_*m*_ and a standard deviation of *σ*=0.1 but is restricted to [0,1]. Initially, an empty pool is created, and *Cr*_*m*_ is set to 0.5. At each iteration, a set of *Cr* values is regenerated and applied to generate the trial vectors, as shown in equation (8). If a trial vector successfully replaces its target vector in the selection process, the corresponding *Cr* value will enter the pool. At the end of each iteration, the new *Cr*_*m*_ is reset as the median of the pool, and then the pool is emptied.

### 3.2. Implementation of MODE in Feature Selection

The proposed MODE is an optimization method over continuous spaces. However, the landscape of feature selection problems is discrete. To implement MODE in feature selection, a binary strategy is incorporated in the proposed method. For a given dataset with *M* features *H*={*h*_1_, *h*_2_,…, *h*_*M*_}, a candidate solution in MODE is represented as

(13) $$\begin{array}{c} X^{\left(i\right)\left(t\right)}=\left(x_{1}^{\left(i\right)\left(t\right)},x_{2}^{\left(i\right)\left(t\right)},\ldots ,x_{M}^{\left(i\right)\left(t\right)}\right),\;x_{j}\in \left[0,1\right], \end{array}$$

where *M* is the number of dimensions of **X**^(*i*)(*t*)^, and it is equal to the dimensionality of the data points. Consequently, a feature subset *S* ⊂ *H* is determined by **X**^(*i*)(*t*)^ and a preset threshold parameter *λ* ∈ (0,1), which is shown in Algorithm 3. This strategy is also employed in the two single-objective methods based on DE.

### 3.3. Three Objectives of the Filter Stage

The first stage of the proposed method is considered a multivariate filter method where the intrinsic characteristics of the raw data are considered. Three objective functions to be minimized are defined in the filter stage to evaluate a feature subset. The first objective function is the number of selected features, and it is considered a prime motivation of feature selection. Previous works [27] have proven that incorporating the number of selected features as an objective is necessary in feature selection. For a given feature subset *S*={*s*_1_, *s*_2_,…, *s*_*n*_}, the first objective function is defined as

(14) $$\begin{array}{c} f_{1}^{\left(filter\right)}=\left|S\right|=n. \end{array}$$

The second objective function strives to select the features with the highest relevance to the target class variable (labeled as malignant or not). This objective aims to maximize the relevance between the features and the target class. Independent of the number of selected features of *S*, it can be defined as follows:

(15) $$\begin{array}{c} f_{2}^{\left(filter\right)}=-D\left(S,c\right)=-\frac{1}{\left|S\right|}\sum_{s_{i}\in S}I\left(s_{i};c\right), \end{array}$$

where *c* is the target class variable and *I*(*s*_*i*_; *c*) is the mutual information between feature *s*_*i*_ and target class *c*.

In addition, the redundancy among each pair of the selected features should be narrowed down because redundant information does little to improve the accuracy of a classifier [43]. The third objective function aims at minimizing the redundancy of the feature subset, and it is defined as follows:

(16) $$\begin{array}{c} f_{3}^{\left(filter\right)}=R\left(S\right)=\frac{1}{{\left|S\right|}^{2}}\sum_{s_{i},s_{j}\in S}I\left(s_{i};s_{j}\right). \end{array}$$

### 3.4. Two Objectives of the Wrapper Stage

The second stage of the proposed method included a wrapper method where the employed classifier should be considered. As shown in Figure 2, a set of nondominated solutions is generated after the filter stage. Although every one of these solutions can be accepted as the starting point of the second stage, it seems more reasonable to select some typical solutions among them according to computational costs. Since the aim of the filter stage is to select a small number of informative features, we select the solution with the smallest number of features as the input of the wrapper stage. Minimizing the classification error rate of a classifier is the main goal of the wrapper stage. In this study, the famous and effective Gaussian NB [44] classifier is applied. The NB classifier is a supervised learning method for classification, which is based on Bayes' theorem and assumes that every pair of features is independent. The Gaussian NB classifier is the state-of-the-art type of NB classifier to handle continuous data in which the continuous values of a special feature are assumed to fit a Gaussian distribution. After selecting a suitable classifier, the two objective functions of the wrapper stage can be defined as follows:

(17) $$\begin{array}{c} \left\{\begin{array}{c} f_{1}^{\left(wrapper\right)}=\text{Num}.\text{ of selected features,}\\ f_{2}^{\left(wrapper\right)}=\text{ErrorRate}. \end{array}\right. \end{array}$$

According to the guidance of Xue et al. [27], the first objective function to be minimized is defined as the number of selected features. In the following content, we can investigate whether it is necessary to take the number of a selected features as an objective in the wrapper stage. Moreover, the average classification error rate of a selected feature subset is defined as the second objective function, which is evaluated by 5-fold cross-validation on the training data. A more detailed description of how the 5-fold cross-validation is performed on training data is given in [45].

### 3.5. Two Single-Objective Feature Selection Methods

Two single-objective feature selection methods based on DE are also proposed in the wrapper stage for comparison. The main difference between the two methods is the choice of fitness functions. The fitness function of one method (DE1) is the same as the second objective function of MODE in the wrapper stage, which is defined as follows:

(18) $$\begin{array}{c} f_{DE1}=\text{ErrorRate}. \end{array}$$

The aim of DE1 is to minimize the classification error rate during the training process. However, the other method (DE2) considers the number of selected features. The fitness function of DE2 is defined as follows:

(19) $$\begin{array}{c} f_{DE2}=\alpha *\frac{\text{Num}.\text{ of selected features}}{\text{Num}.\text{ of all features}}+\left(1-\alpha \right)*\text{ErrorRate}, \end{array}$$

where *α* is a scaling parameter determining the relative importance of the two terms and ErrorRate is the average classification error rate of 5-fold cross-validation on the training data.

To meaningfully devise a fair comparison with the proposed MODE, the procedure of the two DE-based methods is chosen to be similar to that of the proposed MODE mentioned above. The differences between the MODE-based method and the DE-based methods are the selection process and the updating strategy of the external archive. The selection process of the two DE-based methods is the same as the standard DE, as shown in equation (9). The updating strategy of the external archive of the two DE-based methods is based on tournaments with limited size *N*_*a*_.

## 4. Experimental Studies

All the algorithms in this study are implemented in C and Python languages. The programs are executed on a Linux 64-bit system with a 3.4 GHz Core i5 CPU and 8 GB RAM. In addition, the parameters of MODE used in the two stages are listed in Table 1, which have been discussed above. To assess the performance of the proposed two-stage feature selection method, six widely used benchmark microarray datasets are selected in our experiments. The details of these datasets are summarized in Table 2. Note that all of the datasets are binary. The reason for excluding the multiclass datasets is that binary microarray datasets are more common in the field of gene selection [46].

**Figure 3:**
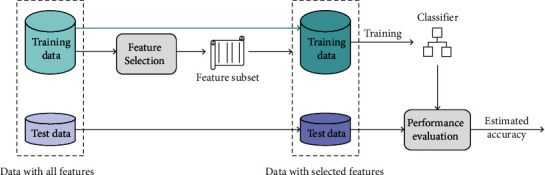
The flowchart of the 5-fold cross-validation experiment.

Because the numbers of samples in microarray datasets are relatively small, 5-fold cross-validation is applied to each dataset to evaluate the effectiveness of feature selection [46]. Specifically, the samples of each dataset are randomly partitioned into five equal subsamples. Four subsamples are used as the training data, and the remaining subsample is used as the test data. Then, the cross-validation process is successively repeated five times. The flowchart of the 5-fold cross-validation experiment is presented in Figure 3. The training data are used by feature selection methods to select a feature subset. Then, the selected feature subset is used to reduce the dimensions of the training data and the test data. Finally, the goodness of the selected feature subset is evaluated by using the test data.

### 4.1. Results of the Filter Stage

The proposed MODE on these benchmark datasets is first implemented in the filter stage. The threshold *λ* is problem-specific and is set properly for each dataset. Since MODE obtains a set of nondominated solutions in each independent run, five independent sets of nondominated solutions with three objectives are generated. We collect five sets of nondominated solutions into a union set and report its statistics in Table 3. It is clear that fruitful solutions are obtained because the values of the three objectives fluctuate significantly. In addition, the small values of |*S*| indicate that few features are selected, and the effectiveness of the filter stage is demonstrated.

**Figure 4:**
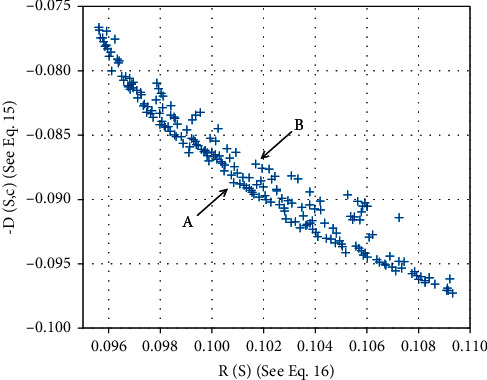
The obtained nondominated solutions (200 data points in one experiment) in the filter stage of the proposed method on the Colon dataset map onto (*R*(*S*); −*D*(*S*; *c*)) space.

Figure 4 shows the nondominated solutions of the Colon dataset in one experiment. These solutions are mapped onto (*R*(*S*); −*D*(*S*; *c*)) space. Similar results can also be obtained for the remaining datasets. Figure 4 shows that *R*(*S*) and −*D*(*S*; *c*) strongly conflict along a curve. This strengthens the rationality of decomposing them as two objectives for optimization. Moreover, a common dominance pattern can be found in Figure 4. For example, the solutions *A* and *B* are nondominated, and *R*(*S*)_*A*_ < *R*(*S*)_*B*_, −*D*(*S*; *c*)_*A*_ < −*D*(*S*; *c*)_*B*_. It is easy to conclude that |*S*|_*A*_ > |*S*|_*B*_. This finding supports our premise that simply reducing the number of features of a subset may diminish its compactness. Therefore, it is necessary to use different criteria to measure the quality of a feature subset.

**Figure 5:**
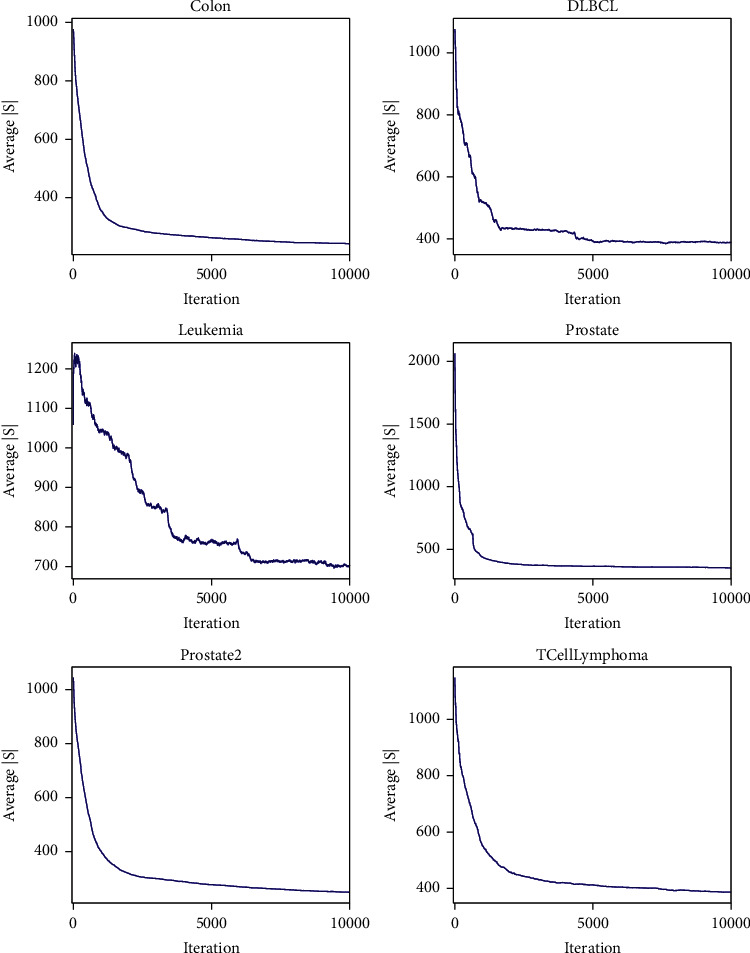
The convergence curves of the average number of selected features of the solutions stored in archive *Arc* in the filter stage.

The first objective |*S*| is used to direct the search procedure and reduce the number of selected features. To observe the changes of the first objective during the evolutionary procedure, Figure 5 shows the convergence curves of the average value of |*S*| of the solutions stored in archive *Arc* for each dataset. We find that the average number of selected features converges quickly and finally stabilizes near a certain value. This means that the filter results are not sensitive to the iteration if the maximum number of iterations *Ite* has been set sufficiently large. In addition, the convergence speed and the stable number of selected features rely on the intrinsic characteristics of each dataset. For example, Leukemia and Prostate have similar scales but converge to different values. Prostate has the largest number of features, but its convergence speed is the fastest.

### 4.2. Results of the Wrapper Stage

Next, the proposed MODE is adopted in the wrapper stage. Inspired by recent works [22, 47], the threshold *λ* is set to 0.5 for all datasets. Finally, five independent sets of nondominated solutions with two objectives are generated. In addition, to analyze the performance of the proposed multiobjective approach, two single-objective approaches mentioned above are executed in the same setting. They also generate five independent sets of solutions for each dataset. Note that for DE2, the classification performance is more important; thus, *α* is set to 0.2 in equation (19).

Since each method generated five sets of nondominated solutions, it will be difficult to compare the performances of these methods. We use the comparison method adopted in previous works [22, 27]. It is worth noting that the classification performance is evaluated and compared on the test data rather than the training data. Specifically, five sets of nondominated solutions that are achieved by the proposed MODE in 5-fold cross-validation are first collected into a union set. Then, the test classification error of each solution is calculated, and the test classification error of the solutions that have the same number of features is averaged. Moreover, the set of “average” solutions is defined as the “average” front. The set of nondominated solutions with the objectives |*S*| and the test classification error in the union set is defined as the “best” front. Furthermore, for the two single-objective methods, we also collect these solutions into a union set, and the same processing method is applied to the union sets. Finally, the performance of the three methods on these three union sets can be compared.

**Figure 6:**
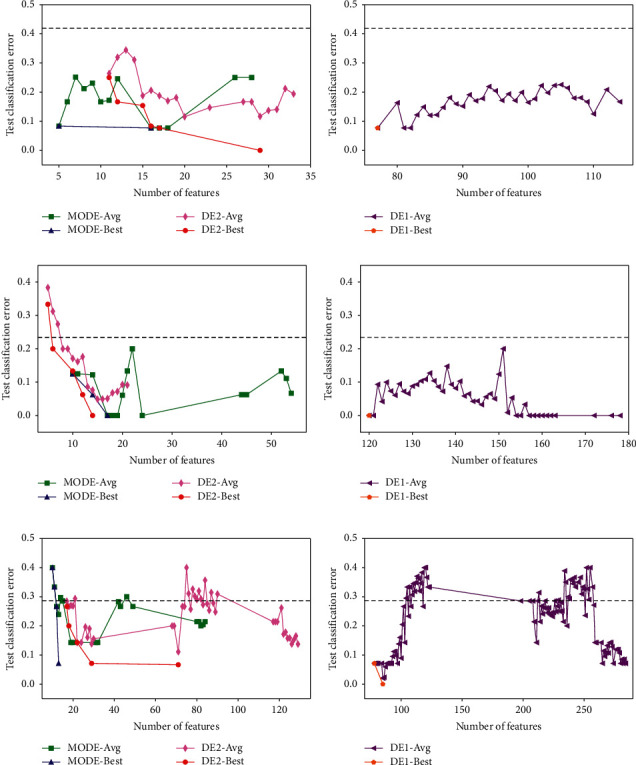
Experimental results of the three methods on the three benchmark datasets ((a) Colon, (b) DLBCL, and (c) Leukemia) in the wrapper stage.

**Figure 7:**
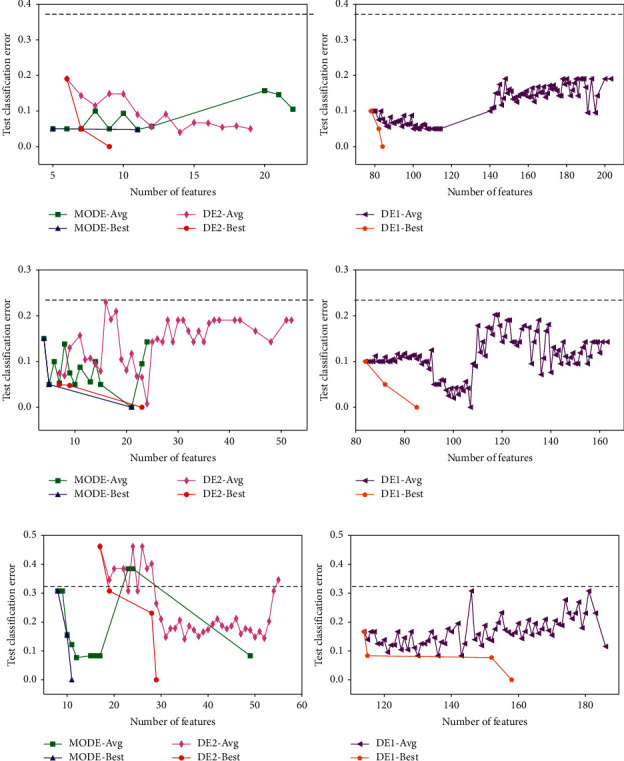
Experimental results of three methods on the three benchmark datasets ((a) Prostate, (b) Prostate2, and (c) TCellLymphoma) in the wrapper stage.

The experimental results of the three methods on the benchmark datasets in the wrapper stage are shown in Figures 6 and 7. The horizontal axis represents the number of selected features of a solution, and the vertical axis represents the test classification error rate. The dashed line crossing each chart represents the average classification error rate of 5-fold cross-validation using all features. Moreover, in each chart, the label “-Avg” in the legends refers to the average front obtained by each method, and the label “-Best” refers to the best front.

According to Figures 6 and 7, the average fronts of the three methods are under the dashed line in most cases. This suggests that all the methods work effectively because their solutions achieve a lower test classification error rate and select fewer features. Moreover, the fluctuation in the curves of the average fronts means that the solutions with a similar number of features can have different test classification error rates. This implies that the feature subset search space is relatively complex.

When we compare DE1 with the other two methods, it is obvious that the classification performance of DE1 is similar to the other two methods on most datasets, but the number of selected features in DE1 is quite larger than that of the other two methods. This is because there is no term in the fitness function of DE1 (equation (18)) that considers the number of selected features. The experimental results strongly suggest the necessity of considering both the classification accuracy of a classifier and the number of features in feature selection.

Both MODE and DE2 consider the number of features in the fitness functions. However, the former method uses a multiobjective technique, while the latter method uses a single-objective technique. As shown in the left charts of Figures 6 and 7, both methods successfully achieve low classification error rates and select fewer features. When we compare these two methods, it can be observed that MODE outperforms DE2. MODE achieves significantly lower test classification error rates on most datasets except Leukemia in terms of the “average” fronts. Furthermore, MODE obtains fewer features. In terms of the “best” fronts, the performance of MODE is also better than that of DE2 because fewer features and a lower test classification error rate are obtained by MODE. Although a fine-tuning parameter *α* in equation (19) can improve the performance of DE2, it requires prior knowledge and should be predefined properly. The results demonstrate the advantage of the proposed MODE in the wrapper stage.

### 4.3. Comparison with Other Methods

To further evaluate the performance of the proposed two-stage method based on MODE, we compare it with seven widely used feature selection methods. GainRatio [48] and ReliefF [49] are two univariate feature selection methods. These methods provide each feature an order ranking according to the relevance between the feature and the target class. We retain the top 10, top 20, and top 40 features to evaluate the performance of these two methods. mRMR [13] is a classical feature method based on mutual information that returns a subset of features with a predefined size. We set the returned number of features to 10, 20, and 40. Correlation-based feature selection (CFS) [50] is also a classical multivariate feature selection method and returns a subset of features. WrapperNB [45] is a wrapper method coupled with the NB classifier. The search strategy of this method is greedy hill climbing augmented with a backtracking facility. In addition, two wrapper methods based on GA and PSO are compared. Based on the parameter settings in the literature [51, 52], the population size *NP* and the maximum iteration *T* of the two methods are set to 50 and 100, respectively. The key parameters of GA are set as follows: the crossover rate *p*_*c*_=0.9, the mutation rate *p*_*m*_=0.1, and the number of elites *N*_*e*_=10. The key parameters of PSO are set as follows: the inertia weight *w*=0.5 and the acceleration constants *c*_1_=1.5,  *c*_2_=1.5.

We use 5-fold cross-validation and follow the workflow in Figure 3 to perform the experiments. The final classifier is the NB classier. To provide a fair comparison, for the proposed method, we select the solutions in the training Pareto front of the union set because the test data cannot be seen until the final performance evaluation. Specifically, the training Pareto front of the union set is constructed according to the training classification performance and the number of features. The comparison is performed on test data, and the results are listed in Table 4. *Acc* represents the average test classification accuracy, and *Gene* represents the number of selected genes (features). As illustrated in Table 4, the proposed method obtains the best classification performance on three (out of six) problems. Moreover, it can select a small number of features and meet the target of gene selection.

We further conduct the Wilcoxon signed-rank test to determine the significant differences between the proposed method and the other methods. The significance level is set to 0.05, and the *p* values are listed in Table 4. It is clear that the proposed method significantly outperforms eight (out of fourteen) methods because the *p* values are smaller than 0.05. In addition, for the remaining six methods, the *p* values are larger than 0.05. This indicates that the proposed method is not significantly better but still obtains competitive results. Therefore, we can conclude that the proposed method can be considered a very competitive method relative to classical methods. The comparison results suggest that the proposed two-stage method based on MODE is a promising method to solve the gene selection problem.

## 5. Conclusion

The gene selection problem is a specific feature selection problem and remains challenging in bioinformatics. In this paper, a two-stage feature selection method was proposed to solve the gene selection problem. The first stage included a multivariate filter method, and the second stage included a wrapper method. Both stages were based on the same MODE but with different objective functions. The objective functions of the filter stage were mainly based on mutual information. The classification error of the NB classifier and the number of selected features were incorporated as the two objective functions in the wrapper stage. In our experiments, six common benchmark datasets were used to test and analyze the performance of the proposed method. In addition, the effectiveness of the proposed method for solving the gene selection problem was verified by comparing it with five classical methods. Since the main differences between the two stages (filter and wrapper) were the objective functions, the proposed method is considered to be an easily understood implementation.

This study provided a new perspective for solving the gene selection problem by using multiobjective optimization because the solution ideas are quite different from the methods based on single-objective optimization. In the future, we plan to apply the proposed method to more gene expression datasets to verify its effectiveness. To improve the performance of the method, the search strategy and the evaluation criteria will also receive sustained attention.

## Figures and Tables

**Algorithm 1:**
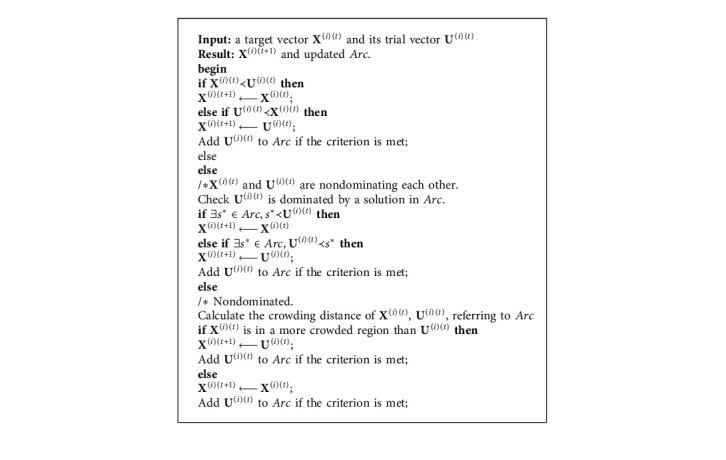
The selection process of the proposed MODE.

**Algorithm 2:**
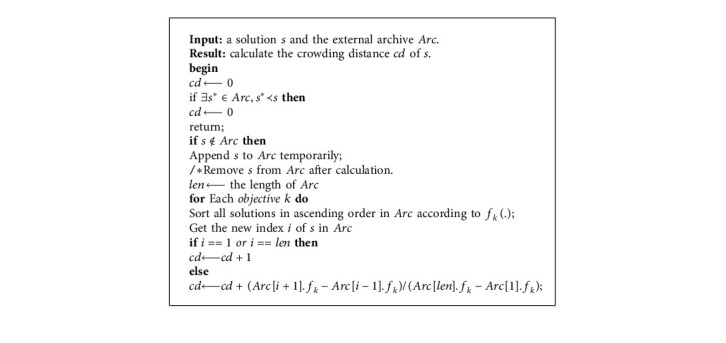
Calculating the crowding distance of a solution.

**Algorithm 3:**
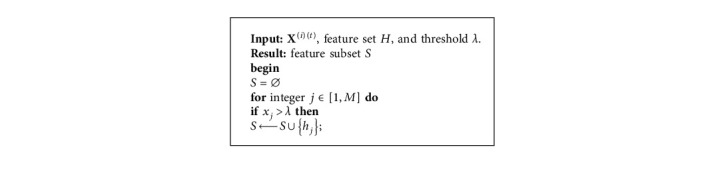
A binary scheme to transform continuous values to binary values for feature selection.

**Table 1:** The parameters in the two stages of the proposed method.

Parameters	Filter stage	Wrapper stage	Description
*NP*	100	50	Population size
*F*	Controlling	Controlling	Mutation scale factor
*Cr*	Self-adapted	Self-adapted	Crossover rate
*N*_*a*_	200	100	The size of archive
*λ*	Tuning	0.5	Threshold for binarization
*Ite*	10000	400	Max number of iterations

**Table 2:** The details of the benchmark datasets.

Dataset	Total Num. of genes (features)	Num. of instances	Num. of classes	Num. of instances for each class
Colon	2000	62	2	40, 22
DLBCL	5469	77	2	58, 19
Leukemia	7129	72	2	47, 25
Prostate	10,509	102	2	52, 50
Prostate2	2135	102	2	52, 50
TCellLymphoma	2922	63	2	43, 20

**Table 3:** The statistical information of the solutions obtained in the filter stage (calculated over the five cross-validation runs).

Dataset		|*S*|	−*D*(*S*, *c*)	*R*(*S*)
Colon	Min	195.0	9.29*E* − 02	−1.03*E* − 01
	Avg ± std	242.3 ± 20.3	1.01*E* − 01 ± 4.00*E* − 03	−8.93*E* − 02 ± 5.88*E* − 03
	Max	300.0	1.11*E* − 01	−7.66*E* − 02
DLBCL	Min	273.0	4.52*E* − 02	−9.95*E* − 02
	Avg ± std	388.9 ± 61.4	5.15*E* − 02 ± 3.46*E* − 03	−8.00*E* − 02 ± 8.20*E* − 03
	Max	554.0	6.03*E* − 02	−6.17*E* − 02
Leukemia	Min	195.0	3.54*E* − 02	−7.18*E* − 02
	Avg ± std	702.1 ± 229.7	4.02*E* − 02 ± 4.61*E* − 03	−5.61*E* − 027.98*E* − 03
	Max	1406.0	6.09*E* − 02	−3.52*E* − 02
Prostate	Min	241.0	6.15*E* − 02	−1.16*E* − 01
	Avg ± std	351.5 ± 51.4	7.19*E* − 02 ± 4.98*E* − 03	−9.52*E* − 02 ± 6.75*E* − 03
	Max	458.0	8.54*E* − 02	−7.92*E* − 02
Prostate2	Min	175.0	1.25*E* − 01	−1.25*E* − 01
	Avg ± std	248.9 ± 54.4	1.49*E* − 01 ± 1.14*E* − 02	−1.04*E* − 01 ± 8.58*E* − 03
	Max	362.0	1.74*E* − 01	−8.86*E* − 02
TCellLymphoma	Min	310.0	5.52*E* − 02	−8.36*E* − 02
	Avg ± std	386.8 ± 30.4	5.94*E* − 02 ± 2.17*E* − 03	−7.29*E* − 02 ± 4.67*E* − 03
	Max	469.0	6.56*E* − 02	−6.22*E* − 02

**Table 4:** The comparison results of different methods on the six benchmark datasets.

		Colon	DLBCL	Leukemia	Prostate	Prostate2	TCell Lymphoma	*p* value
All features	Acc	58.08%	76.58%	71.33%	62.81%	76.57%	67.82%	0.000 011
	Gene	2000.0	5469.0	7129.0	10,509.0	2135.0	2922.0	

The proposed method	Acc	**88.06%**	87.67%	72.10%	**94.41%**	90.90%	**84.47%**	—
	Gene	6.2	12.7	12.4	10.0	7.0	10.1	

GainRatio	Acc	79.10%	83.08%	62.67%	91.24%	**93.19%**	74.87%	0.005 106
	Gene (top 10)	10.0	10.0	10.0	10.0	10.0	10.0	
	Acc	78.85%	87.08%	71.14%	92.24%	92.19%	77.95%	0.054 282
	Gene (top 20)	20.0	20.0	20.0	20.0	20.0	20.0	
	Acc	82.31%	87.08%	64.10%	91.24%	92.14%	73.21%	0.015 658
	Gene (top 40)	40.0	40.0	40.0	40.0	40.0	40.0	

ReliefF	Acc	82.44%	88.42%	69.62%	92.19%	92.14%	76.28%	0.362 370
	Gene (top 10)	10.0	10.0	10.0	10.0	10.0	10.0	
	Acc	82.44%	88.33%	72.38%	92.24%	90.14%	76.15%	0.452 807
	Gene (top 20)	20.0	20.0	20.0	20.0	20.0	20.0	
	Acc	83.97%	84.50%	69.90%	92.24%	92.19%	74.62%	0.236 936
	Gene (top 40)	40.0	40.0	40.0	40.0	40.0	40.0	

mRMR	Acc	85.38%	82.92%	58.76%	89.33%	90.24%	74.74%	0.016 822
	Gene	10.0	10.0	10.0	10.0	10.0	10.0	
	Acc	83.97%	85.67%	68.38%	92.24%	92.24%	68.46%	0.037 739
	Gene	20.0	20.0	20.0	20.0	20.0	20.0	
	Acc	82.31%	86.92%	**75.33%**	93.24%	91.19%	70.13%	0.180 025
	Gene	40.0	40.0	40.0	40.0	40.0	40.0	

CFS	Acc	82.18%	**92.08%**	68.48%	93.19%	91.19%	72.95%	0.456 408
	Gene	26.8	92.0	90.0	78.8	33.8	44.0	

WrapperNB	Acc	76.15%	76.58%	60.00%	91.14%	88.33%	69.87%	0.002 557
	Gene	6.2	4.2	6.6	5.2	5.4	6.2	

GA	Acc	71.03%	76.67%	70.00%	63.81%	86.33%	69.49%	0.000 114
	Gene	122.2	378.0	514.8	803.2	105.6	161.8	

PSO	Acc	65.90%	81.92%	68.29%	71.67%	82.48%	71.03%	0.000 026
	Gene	134.0	371.6	455.6	506.2	115.2	150.4	

The best classification accuracy for each benchmark dataset is in bold.

## Data Availability

The data used to support the findings of this study are included within the article and can be obtained from the corresponding authors upon request.
